# Assessment of Short-Term Outcomes of Total Knee Arthroplasty Performed With and Without a Tourniquet

**DOI:** 10.7759/cureus.25324

**Published:** 2022-05-25

**Authors:** Kuldip Sandhu, Deepak Goyal, Karamdeep S Kahal, Harish Khichy

**Affiliations:** 1 Department of Orthopaedics, Government Medical College, Patiala, IND

**Keywords:** quadriceps function, tourniquet, range of motion, total knee arthroplasty, pain

## Abstract

Background: Tourniquet use has been advocated for better visualization of the surgical field as it exsanguinates most of the blood from the limbs to the central compartment. On the contrary, its use may increase postoperative pain and recovery of quadriceps function, thereby increasing the length of stay (LOS). The study aims to assess short-term outcomes of total knee arthroplasty (TKA) performed with and without a tourniquet.

Methodology: Eighty-six patients scheduled for TKA either with or without a tourniquet were selected and divided into two groups using simple random sampling. Knee replacements were performed with a tourniquet in group I and without a tourniquet in group II. In all cases, blood loss was estimated. A visual analog scale (VAS) was used for the assessment of postoperative pain. In the study, range of motion (ROM) and quadriceps lag were also assessed on postoperative day 2 and discharge.

Results: There were 23 (26.7%) males and 20 (23.2%) females in group I and 28 (32.5%) males and 15 (17.4%) females in group II (p = 0.07). On comparing mean age and body mass index (BMI), statistically insignificant results were obtained. In group I and group II, a statistically significant difference was obtained in the estimation of mean total blood loss as 780.4 ± 152.49 and 1146.2 ± 193.14 ml, respectively (p = 0.02). Neither on postoperative day 2 nor at the time of discharge, no significant results were obtained in observing the ROM at the knee joint and quadriceps lag.

Conclusion: It was found that tourniquet use is associated with lower blood loss and similar postoperative pain, ROM, quadriceps lag, and LOS.

## Introduction

End-stage knee osteoarthritis is well managed with the procedure of total knee arthroplasty (TKA), which is a commonly performed surgical technique by orthopedic surgeons. It has been proven that the outcome of this method is comparatively better than conservative treatment options [1]. The cases of end-stage knee osteoarthritis have alarmingly increased in the last few years and subsequently the number of TKA procedures [2]. This method proved to decrease the hospital stay and accelerate ambulation and rehabilitation with no increased adverse events [3].

There is a contradictory suggestion about pain, loss of blood, and treatment outcomes with different uses of tourniquets. It is evident that tourniquet use promotes better surgical field assessment, superior cement attachment, and reduced patient blood loss [4]. On the other hand, some authors found increased pain, ischemia of the lower limb, late range of motion (ROM) and recovery of function of quadriceps, and faster quadriceps sarcopenia in the geriatric population. Some researchers advocated increased thromboembolism, enhanced hospital stay, and a rise in hospital readmission with tourniquet use [5].

The procedure of TKA is mostly done with or without the use of a tourniquet either using spinal anesthesia (SA) or general anesthesia (GA) [6]. With the use of SA or GA, there are fewer chances of death, complications, requirements for ICU treatment, a necessity for blood transfusions, and length of stay. It is also observed that the use of a tourniquet can also affect the treatment outcome in patients undergoing TKA [7,8]. In this study, we performed a prospective study to assess short-term outcomes of TKA performed with and without a tourniquet.

## Materials and methods

A sum total of 86 patients undergoing TKA with or without tourniquet were selected. All gave their written consent for the participation, and the study was approved by the Institutional Ethical Committee of Government Medical College, Patiala, with a vide letter no. (Trg).EC.NEW/INST/2020/997/28387. 

Demographic data were recorded in case history proforma. All cases with features of end-stage knee osteoarthritis were included and operated by a single orthopedic surgeon. However, the patients with a history of traumatic or rheumatic arthritis of the knee, who did not give consent to be a part of the study, with low-grade osteoarthritis of the knee, American Society of Anesthesiologists (ASA) class 3 or more (uncontrolled hypertension or diabetes mellitus), or patients posted for bilateral TKA were excluded from the study. The patients were divided into two groups using simple random sampling. In group I, knee replacements were performed with a tourniquet, and in group II, the same procedure was performed without a tourniquet. In all cases, blood loss was estimated. With a visual analog scale (VAS), postoperative pain was assessed. If VAS is "0," then it is designated as "no pain," and if VAS is "10," then it was titled as being the "worst pain." Inj. Tramadol 50 mg was administered intravenously if the patient complains of pain with VAS > 4. The ROM and quadriceps lag were determined by a physiotherapist with a goniometer on the second day after surgery and discharge. The patient was discharged on day 5 after the completion of intravenous medications (institutional protocol).

After preoperative preparation, combined spinal and epidural anesthesia in a sitting position was administered to all the patients; 2.75 ml of 0.5% inj. Bupivacaine was injected in the intrathecal space, and a catheter was secured in the epidural space. The patients were immediately lied down, and hemodynamic variables (heart rate, systolic/diastolic blood pressure, and temperature) were monitored throughout the procedure by a trained anesthetist. 

Estimation of blood loss

The blood loss was estimated by the following three steps [9]:

Step 1

Patient's blood volume (PBV) is calculated using the formula, PBV = (k1 x h^3^) + (k2 x w) + k3, where PBV = patient’s blood volume in liters (L), h = height of the patient in meters (m), w = weight of the patient in kilograms (kg), k1 = 0.3669 for men and 0.3561 for women, k2 = 0.03219 for men and 0.03308 for women, and k3 = 0.6041 for men and 0.1833 for women. After obtaining it, PVB was then converted to milliliters.

Step 2

The volume of RBC loss was estimated as RBC = PBV x (Hct_preop_ - Hct_postop_), where RBC = red blood cell volume loss in milliliters (mL), Hct_preop_ = preoperative hematocrit, and Hct_postop_ = postoperative hematocrit. 

Step 3

Finally, the RBC loss was converted to total blood volume loss (TBV) as TBV = RBC/Hct_avg _+ Number of units of blood transfused/Hct_avg_, where Hct_avg_ = Hct_preop_ + Hct_postop_/2. The idea behind the calculation of TBV is that due consideration was done to the transfused blood, and the RBC was divided by the average hematocrit. An average unit of blood contains 250 mL with a Hct of 0.8, which is the red cell equivalent of 168 mL with a Hct of 1. As we know that this is diluted by the patient’s blood volume, the final value must in turn be divided by the average hematocrit.

Statistical analysis

All statistical analyses were performed using the IBM Statistical Package for the Social Sciences (SPSS) Statistics for Windows, Version 20.0 (IBM Corp., Armonk, NY). The parametric data were compared by the student's t-test while non-parametric data were compared using the chi-square test. A p-value < 0.05 was considered significant.

## Results

The study involved 86 patients, and all patients had successfully completed the study. There were 23 (26.7%) males and 20 (23.2%) females in group I and 28 (32.5%) males and 15 (17.4%) females in group II (p = 0.07). The mean age of patients was 68.72 ± 6.91 and 70.28 ± 7.41, respectively (p = 0.82). On comparing the BMI of the patients in both groups, a statistically insignificant difference was obtained (p = 0.23) (Table 1).

**Table 1:** Demography of the patients Data presented as number (percentage) or mean ± SD.

Variables	Group I (with tourniquet)	Group II (without tourniquet)	P-values
Male:Female	23 (26.7%):20 (23.2%)	28 (32.5%):15 (17.4%)	0.07
Age (years)	68.72 ± 6.91	70.28 ± 7.41	0.82
BMI (kg/m^2^)	28.86 ± 3.29	29.97 ± 4.83	0.23

In group I and group II, a statistically significant difference was obtained in the estimation of mean total blood loss as 780.4 ± 152.49 and 1146.2 ± 193.14 ml, respectively (p = 0.02); however, on comparing the mean length of stay, the results were statistically insignificant as 4.86 ± 0.91 and 4.15 ± 0.89 days in group I and group II, respectively (p = 0.65), and a similar comparable difference was obtained in mean pain scores of both the groups (p = 0.71) (Table 2).

**Table 2:** Assessment of parameters *p < 0.05; data presented as Mean ± SD.

Parameters	Group I (with tourniquet)	Group II (without tourniquet)	P-values
Total blood loss (ml)	780.4 ± 152.49	1146.2 ± 193.14	0.02*
Length of stay (days)	4.86 ± 0.91	4.15 ± 0.89	0.65
Pain score	4.31 ± 1.21	4.72 ± 1.39	0.71

On day 2 of the postoperative day, the ROM at the knee during extension was observed to be 7.54 ± 1.21 degrees in group I and 6.12 ± 0.98 degrees in group II (p = 0.12). The ROM on the same day during flexion was 76.2 ± 1.19 degrees and 78.6 ± 1.28 degrees in patients with or without a tourniquet, respectively (p = 0.53). On the same day, the quadriceps lag was 19.3 ± 1.14 degrees and 20.1 ± 1.62 degrees in group I and group II, respectively (p = 0.31) (Table 3). Similar, insignificant results were obtained at the discharge of the patients by comparing ROM at knee and quadriceps lag between the cohorts (Table 3).

**Table 3:** Assessment of knee (range of motion) and quadriceps lag Data presented as Mean ± SD. ROM: Range of motion.

Knee ROM	Group I (with tourniquet)	Group II (without tourniquet)	P-values
Day 2 extension (degrees)	7.54 ± 1.21	6.12 ± 0.98	0.12
Day 2 flexion (degrees)	76.2 ± 1.19	78.6 ± 1.28	0.53
Day 2 lag (degrees)	19.3 ± 1.14	20.1 ± 1.62	0.31
Discharge extension (degrees)	7.35 ± 1.39	5.84 ± 1.03	0.09
Discharge flexion (degrees)	88.4 ± 2.02	85.2 ± 1.83	0.13
Discharge lag (degrees)	15.4 ± 1.82	16.8 ± 1.72	0.45

## Discussion

In the current scenario, TKA protocols work on fast track mode, and thereby, it focuses on the reduction in the length of stay (LOS) and speeds up the ambulation and general rehabilitation without compromising the health of the patients [9,10]. Still, many patients after TKA complains of acute or chronic postoperative pain, which ultimately creates an impact on the rehabilitation and recovery of the patients. Postoperative pain is a common problem having an incidence of 10%-36%, thereby leading to a major cause of dissatisfaction in patients following TKA [11]. In addition to it, concerns about the long duration of opioid use have emphasized the need for more structured studies on the need for better and enhanced postoperative pain management [12,13]. In the present study, short-term outcomes of TKA were assessed in patients performed with and without a tourniquet.

Our results showed that there were 23 (26.7%) males and 20 (23.2%) females in group I and 28 (32.5%) males and 15 (17.4%) females in group II. Grigoras et al. in their study on 97 patients undergoing TKA with or without a tourniquet divided patients into two equal groups. One group was a non-tourniquet group, and the other was the tourniquet group [13]. The tourniquet group experienced one thromboembolic event, but the thromboembolic index of suspicion did not differ. The amount of blood loss was 32% more in the non-tourniquet group compared to the tourniquet group. Both groups were comparable in terms of pain, ROM, LOS, and quadriceps lag on day 2 and at discharge. Females had a significantly lower day 2 maximum flexion than males in the tourniquet group. They concluded from their study that the use of a tourniquet is associated with a lesser amount of blood loss and similar quadriceps lag, postoperative pain, LOS, ROM, and thromboembolic risk [13]. Furthermore, both genders may show variation in tolerating tourniquet, but females revealed worse short-term outcomes as compared to the male population.

Our results showed that in comparison to total blood loss in both groups, 780.4 ± 152.49 and 1146.2 ± 193.14 ml were found in groups I and II, respectively (p = 0.02), mean LOS was 4.86 ± 0.91 and 4.15 ± 0.89 days in groups I and II (p = 0.65), and the comparable difference was obtained in mean pain scores of both the groups (p = 0.71). Alcelik et al. reported a paradoxical higher blood loss in a group when the combination of a tourniquet and tranexamic acid was used as compared to tranexamic acid alone in another group [14].

We found that group I and group II on day 2 extension had values of 7.54 ± 1.21 degrees in group I and 6.12 ± 0.98 degrees in group II; on day 2, flexion was 76.2 ± 1.19 degrees and 78.6 ± 1.28 degrees; on day 2, quadriceps lag was 19.3 ± 1.14 degrees and 20.1 ± 1.62 degrees; discharge extension value was 7.35 ± 1.39 and 5.84 ± 1.03 degrees; discharge flexion was 88.4 ± 2.02 and 85.2 ± 1.83 degrees, and discharge lag at quadriceps was 15.4 ± 1.82 and 16.8 ± 1.72 degrees, respectively. Goel et al. divided 200 patients undergoing TKA into two groups of 100 each. One group used a tourniquet (100), and the second group did not use a tourniquet (100) [15]. Parameters such as functional assessment testing with timed up and go (TUaG) test and VAS pain scores [16] were recorded. Apart from this, stair-climb tests, blood loss, surgical field visualization, and ROM were also recorded in both groups. It was observed that calculated blood loss was 1,148.02 mL in the no-tourniquet group as compared to the tourniquet group with 966.64 mL blood loss. Similarly, there was more difficulty with surgical field visualization in the no-tourniquet group. The tourniquet group had greater knee extension at the first follow-up [16].

Tai et al. included 72 patients undergoing TKA in their study [16]. They were also divided into two groups of 36 each. The authors assessed the amount of total blood loss and changes in C-reactive protein (CRP) and preoperative and postoperative levels of creatine phosphokinase on days 1, 2, and 4. The authors further evaluated the record of pain at the thigh/knee, swelling at the limb, and hospital stays in the case history performa. Tourniquet group patients demonstrated less hemoglobin with 2.6 g/dL, non-tourniquet group patients showed hemoglobin of 3.7 g/dL, hematocrit value was 7.6% as compared to 10.4%, less calculated blood loss, i.e., 303 mL as compared to 423 mL, minimal increases in C-reactive protein with a value of 175 mg/dL versus 139 mg/dL, and creatine phosphokinase with a value of 214 U/L versus 162 U/L in the non-tourniquet group [16]. No-tourniquet group patients exhibited less postoperative pain. Parameters such as swelling, rehabilitation progress, or hospital stays showed no difference in either group.

Limitations

The study involves a small sample size; a large sample size may advocate better results. The study did not include the thromboembolic risk of the patients similar to the studies by other researchers. Since the study was conducted in a hospital that is taking care of a poor population, they cannot afford CT angiography for evaluation of thromboembolic risk. Third, the study did not evaluate the short-term outcomes if the comparison was done between males and females. In such situations, how different gender behaves with or without the use of a tourniquet can be evaluated.

## Conclusions

The study advocates that the use of a tourniquet during TKA limits total blood loss. This is particularly helpful in cases having cardiopulmonary comorbidities as such types of patients cannot bear huge blood losses. However, no differences were found in early postoperative functional outcomes in terms of ROM at the knee joint, quadriceps lag, and pain scores when compared to the patients whose surgery was performed without a tourniquet.
